# Value of projectional imaging relative to cross-sectional imaging to assess catheter tip position in the superior vena cava: evaluation of reader variability

**DOI:** 10.1093/bjr/tqae218

**Published:** 2024-10-29

**Authors:** Giuseppe Gullo, David Christian Rotzinger, Pierre Frossard, Anaïs Colin, Guillaume Saliou, Salah Dine Qanadli

**Affiliations:** Department of Diagnostic and Interventional Radiology, University Hospital, 1011 Lausanne, Switzerland; Faculty of Biology and Medicine (FBM), University of Lausanne (UNIL), 1015 Lausanne, Switzerland; Department of Diagnostic and Interventional Radiology, University Hospital, 1011 Lausanne, Switzerland; Faculty of Biology and Medicine (FBM), University of Lausanne (UNIL), 1015 Lausanne, Switzerland; Department of Diagnostic and Interventional Radiology, University Hospital, 1011 Lausanne, Switzerland; Department of Diagnostic and Interventional Radiology, University Hospital, 1011 Lausanne, Switzerland; Department of Diagnostic and Interventional Radiology, University Hospital, 1011 Lausanne, Switzerland; Faculty of Biology and Medicine (FBM), University of Lausanne (UNIL), 1015 Lausanne, Switzerland; Department of Diagnostic and Interventional Radiology, University Hospital, 1011 Lausanne, Switzerland; Faculty of Biology and Medicine (FBM), University of Lausanne (UNIL), 1015 Lausanne, Switzerland

**Keywords:** central venous catheters, observer variation, consensus, vena cava, superior, radiography, thoracic, tomography scanners, X-ray computed

## Abstract

**Objectives:**

The cavo-atrial junction (CAJ) is the most appropriate central venous catheters CVC tip location to reduce complications. Among chest X-ray (CXR) landmarks for tips assessment, only the pericardial reflection lies in the same plane as the vascular structures. We aimed to evaluate the observer variability to determine tip positioning on CXR, using CT as a gold standard.

**Methods:**

We retrospectively analyzed 107 CT scans of patients with port catheters (January–December 2021). The tip to CAJ distance (DCAJ) was measured on both projectional (PJ) and cross-sectional (CS) CT images by 2 × 2 observers (within and between evaluations). Observational statistics included paired t-tests, repeatability coefficients (RC), and intraclass correlation coefficients (ICC), with data visualized using Bland-Altman plots.

**Results:**

All ICC were >0.9, indicating excellent reliability. The mean difference between observers comparing CS and PJ was 0.13 ± 0.80 cm (*P* = .10) with outer 95% confidence limits of 1.92 cm and −2.17 cm and an RC of 1.79 cm.

**Conclusion:**

CXR provides a reliable method for CVC tip localization, though assessment variability is ±2 cm.

**Advances in knowledge:**

CXR assessment of CVC tips shows both intra- and inter-individual variability, due to challenges in identifying the CAJ and catheter tip . While considering the 3 cm anatomical zone around the CAJ acceptable, operators should be aware of the 2 cm variability resulting from CXR assessment. To account for this variability and avoid the risk of positioning the tip beyond 3 cm from the CAJ, operators should reduce the CXR-based acceptable zone to 1 cm around the CAJ, impacting approximately 30% of procedures.

## Introduction

For 50 years, assessing correct central venous catheter (CVC) positioning has been a primary concern to avoid complications. Incorrect CVC tip positioning can lead to serious complications such as vessel perforation, thrombosis, pneumothorax, pericardial tamponade, or arrhythmia and may present fatal issues.^1^ During this period, chest X-ray (CXR) has been widely used for CVC positioning assessment.^2^

Other direct assessment technologies, such as transesophageal echocardiography (TEE), also exist, but the use of TEE in daily practice remains limited due to its invasiveness.^3^ Additionally, transthoracic echocardiography is limited by the operator’s experience.^4^ ECG-guided positioning is also possible and noninvasive, but allows only an indirect assessment of the catheter location, which may be interpreted as being correctly positioned while in the wrong position.^5^ None of these technologies has gained complete consensus. On the other hand, CXR remains the assessment method considered the standard of reference by many physicians,^6^ though it is sometimes criticized for the challenges in accurately estimating catheter tip location.^7^

In 1998, the National Association of Vascular Access Networks published a position statement regarding terminal tip placement of central catheters, establishing the junction of the superior vena cava (SVC) with the right atrium (RA) as the most appropriate location.^8^ More than 20 years later, the cavo-atrial junction (CAJ) is still considered the central vascular access device tip location with the best safety profile in adults and children.^9^

Different CXR landmarks have been proposed for the assessment of tip position relative to the CAJ, such as the bronchus and trachea,^10^ thoracic vertebrae,^2^ clavicle,^11^ costal arches,^12^ and pericardial reflection.^10^ Except for the last, all of these landmarks rely on structures independent from central venous circulation. That makes their use highly questionable, as they are not located in the same plane as the vascular structures they are used to assess. These discrepancies led to a review article in the 2000’s pointing to the controversy on the appropriate position of CVC.^7^ Twenty years later, despite technological advances in X-ray imaging, challenging CRX regarding tip position remains a recurring question.^13^^,^^14^

Thus, to determine the intrinsic capacity of CXR to assess tip position, we aimed to evaluate the observer variability in accurately depicting CVC tip positioning on CXR while using CT images as a gold standard.

## Methods

The protocol of this retrospective observational study was approved by the local Ethics Commission (BASEC ID 2022-01221). The patients included in this study provided written informed consent for re-use of clinical data.

### Study design

Tip positioning was assessed by measuring the distance (positive or negative) from the CVC tip to the CAJ, referred to as the distance from tip to CAJ (DCAJ). Different observers measured the DCAJ on projectional imaging (PJ) compared with cross-sectional imaging (CS), which served as a reference standard. The intra/interobserver variation/agreement between PJ and CS determined the capability to consider PJ as a valuable technique for tip positioning.

Our study used CT as the gold standard modality for tip position identification because the scientific design required an independent reference. However, the routine use of CT in clinical practice is obviously unsuitable due to the associated cost, radiation dose, and availability of the technology.

CXR effective dose is generally accepted as 0.015 mSv for a posteroanterior projection.^15^ In comparison, CT may range from 0.7 mSv for routine lung nodules follow-up to 3.8 mSv for standard diagnostic exams,^16^ representing a 50 to 250-fold dose increase.

Regarding availability, we consider a 40-minute delay for non-scheduled CXR to be obtained (mean time in our institution), while CT waiting time is about 150 minutes if given a high priority and depending on the daily schedule. Obviously, a request for tip verification on CT would be considered a last-in-line indication, and delays would vastly exceed the aforementioned time limit.

### Participants and setting

The studied population comprised adults who underwent a CT scan covering the thorax in the Lausanne University Hospital (CHUV) between January 1, 2021 and December 31, 2021.

We retrospectively screened consecutive patients who underwent CT for oncologic follow-up. The inclusion criteria for this study were adult patients (≥18 years old) who signed the CHUV general consent form for research and were each wearing a port access catheter (PAC) device.

Exclusion criteria were a technically inadequate examination (eg, topogram artifacts, respiratory artifacts, motion artifacts), noncorresponding level of the CVC tip between PJ and CS imaging (the tip position was confirmed on axial CT images and cross-referenced with the scout topogram through a line representing the axial CT image level), nonidentifiable CVC tip on PJ, and absence of consent.

### Data sources and variables

Patient demographics (age, sex) and radiological data (DCAJ) were collected from electronic medical record systems and measured on a picture archiving and communication system (PACS), respectively.

#### Imaging technique

CT examinations were performed on 256-detector CT scanners (Revolution CT and Revolution Apex, GE Healthcare, Milwaukee, WI). CT scanner detector coverage was 80 mm, and images were reconstructed at 1.25-mm thickness with 1-mm intervals or 2.5-mm thickness with 2-mm intervals using a 50-cm field of view. Gantry rotation times were 0.28 and 0.35 seconds, and the scan pitch was about 0.5 (depending on dose adjustment and if a dual-energy protocol was applied). Depending on patient habitus, the kVp varied between 80 and 140, and the maximum tube current ranged from 120 to 840 mA.

For topograms, the kVp range was 80-140 and tube current ranged from 10 to 660 mA.

#### Measurements definition

**Figure 1.**
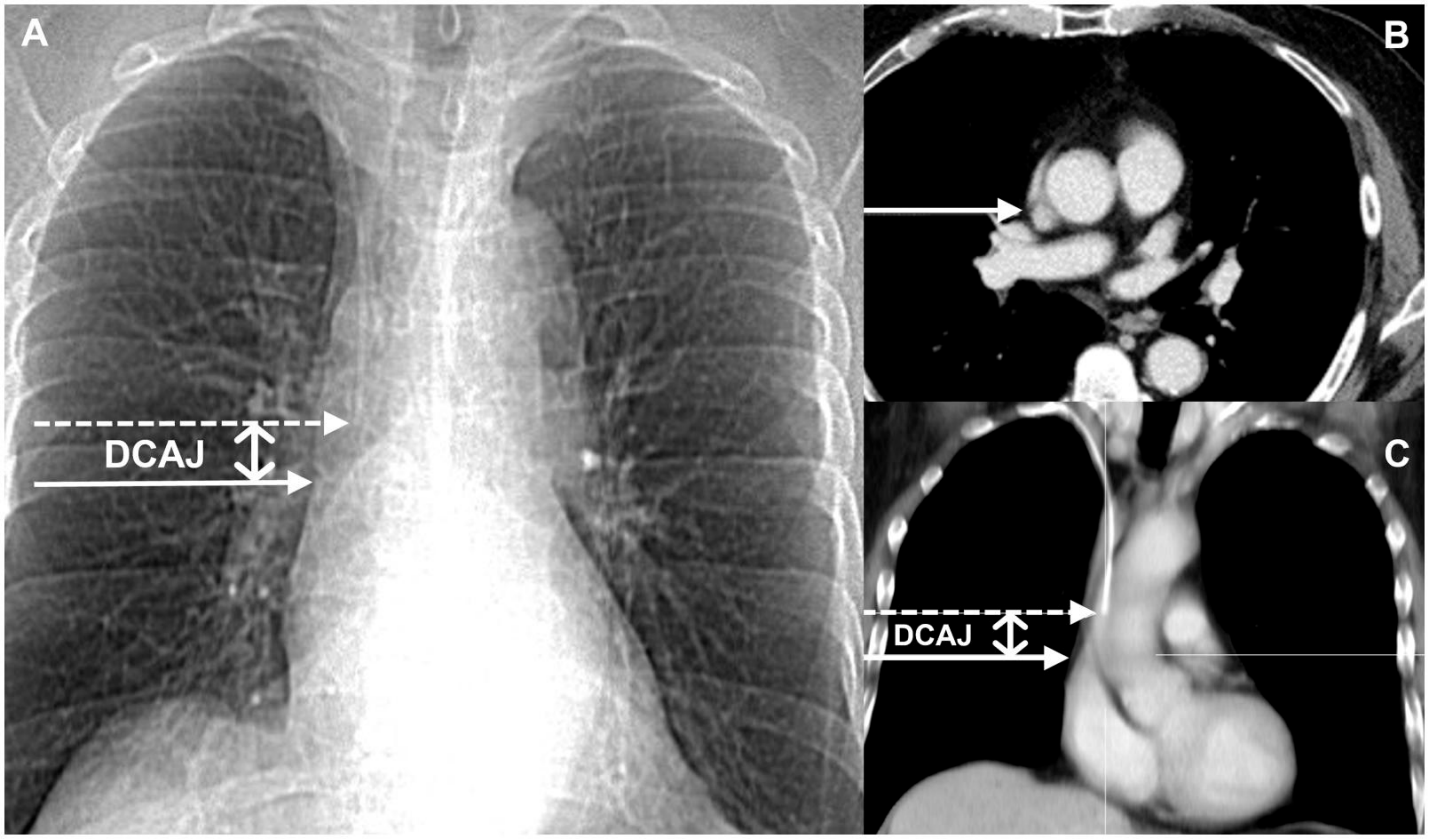
(A) Projectional CT image. Intersection between the right lateral margin of the superior vena cava (SVC) and the superior border of the right atrium determines the level of cavo atrial junction (CAJ) (closed arrow). The catheter tip is identified in the SVC (dashed arrow). (B) Axial CT image. Fat-filled (hypodense) band (closed arrow) determines the crista terminalis and thus the level of CAJ. (C) Multiplanar CT reconstruction. The plane of the axial image containing the CAJ is reported on the multiplanar reconstruction (closed arrow). The catheter tip is identified in the SVC (dashed arrow). (A + C) DCAJ is the vertical distance from the catheter tip to the CAJ (double open arrow).

The DCAJ was measured on both the projectional image (PJ) and the axial image (CS) (Figure 1).

On PJs, the CAJ was considered to be located at the intersection of the right lateral margin of the SVC and the superior border of the RA (cardiac silhouette) (Figure 1).^17^

On CSs, the CAJ was considered to be located at the level corresponding to the crista terminalis. The location of the crista terminalis (from the epicardial surface) was identified as a subtle fat-filled groove called the sulcus terminalis in which the sinoatrial node is located.^18^ The DCAJ was interpolated by toggling between axial sections and multiplanar reconstructions (Figure 1).^3^

DCAJ was subsequently used to categorize tip position into 3 groups based on work published by Gullo et al.^19^

T1 (optimal position): the tip of the catheter lies within 1 cm of the CAJ. T2 (suboptimal position): the tip of the catheter lies within 1-3 cm of the CAJ. T3 (need to replace position): the tip of the catheter lies more than 3 cm distant from the CAJ.

#### Image analysis

Tip position was assessed independently by radiologists and radiology technicians, all of whom are members of the cardiothoracic and vascular unit, with a minimum experience of 6 years in catheter tip reading. Projectional image readers were blinded to CS and multiplanar reconstructions, while cross-sectional readers were blinded to topogram imaging. The interpretation was standardized during a joint training session with a set of patient images (not included in the study) on a PACS workstation (Carestream Health, Rochester, New York, United States, V12.2.8.100). Images were evaluated using a BARCO diagnostic monitor (Barco Nio 5MP MDNG-5221 LED monitor; Barco, Kortrijk, Belgium). Disagreements were resolved by a consensus reading in an additional joint reading session.

**Figure 2.**
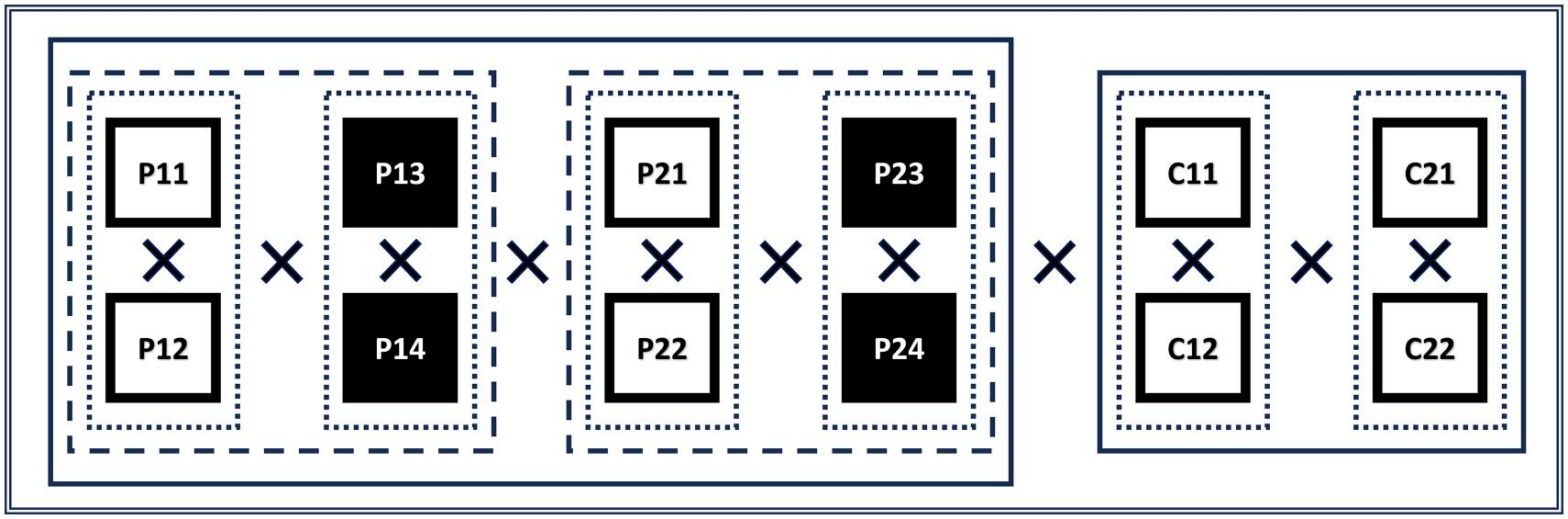
Comparison matrix: P: projectional readers/C: cross-sectional readers. Dotted line: intra-observer comparison. Dashed line: normal vs. inverted greyscale, intra-observer comparison (mean of two observations). Solid line: projectional vs. projectional and cross-sectional vs. cross-sectional, inter-observer comparison (mean of 4 observations respectively mean of 2 observations). Double solid line: projectional vs. cross-sectional, inter-observer comparison (mean of 8 observations respectively 4 observations).

- Projectional image readers 1 and 2 each interpreted 4 observations (projectional/reader no./observation nos. P11, P12, P13, and P14; and P21, P22, P23, and P24, respectively) of tip position on the full set of TP images separated by at least 1 month to make sure that the earlier measurements were not remembered. The first 2 observations were white-on-black imaging (white = bones), and the last 2 were black-on-white imaging (black = bones). Grayscale inversion was performed through the built-in PACS software (Figure 2).
- Cross-sectional image readers 1 and 2 each interpreted 2 observations (cross-sectional/reader no./observation nos. C11, C12, C21, and C22) of tip position on the full set of CT images separated by at least 1 month to avoid recall bias (Figure 2).

### Sample size and statistical methods

The sample size estimation was designed for 80% power and a 5% type I error rate on the basis of a paired comparison (paired *t*-test).

Sample size was calculated with R data analysis software (R version 4.0.4 (2021-02-15)—“Lost Library Book”). The considered difference between PJ and CS for final catheter tip position (DCAJ) was 5 mm (differences of up to 5.2 mm in CT intraobserver measurement have been documented^20^ with a SD of 1.7 (based on our own clinical expertise).

This yielded a cohort of at least 93 participants.

The statistical analysis of mean DCAJ measurements was performed using the Wilcoxon signed-ranks test or a paired *t*-test, as appropriate. A *P* value <.05 was considered statistically significant. The intraclass correlation coefficient (ICC) allowed the determination of intra-interrater agreement on continuous measurements.

Bland-Altman agreement analysis was also performed (mean of the differences [bias]), with limits of agreement (LoA) containing 95% of differences between repeated measurements and repeatability coefficients (RCs) directly related to the LoA (the value below which the absolute differences between 2 measurements would lie with 95% probability).

## Results

### Participants

**Figure 3.**
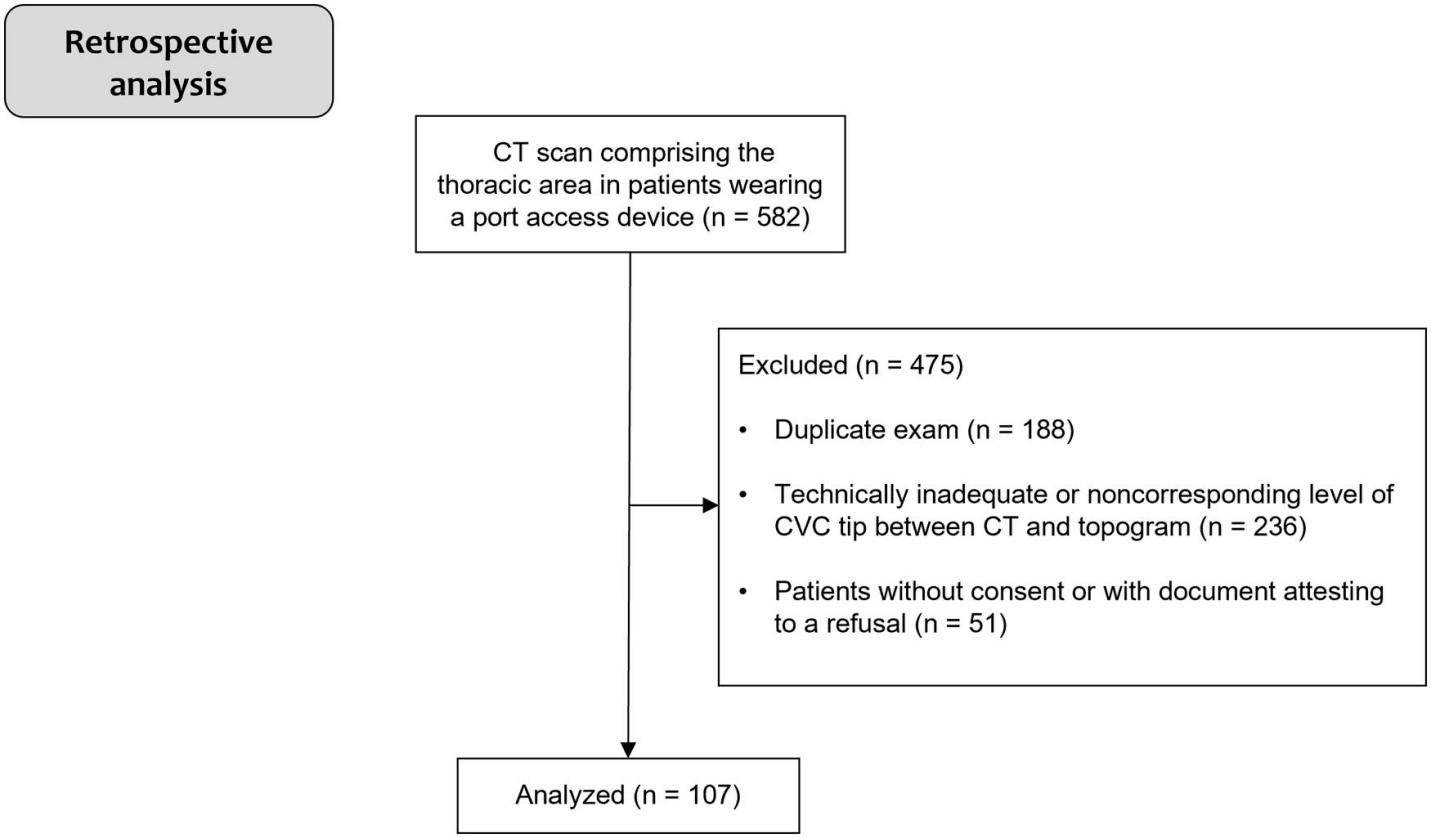
Study flow chart of participant selection.

A total of 582 CT scans were retrospectively reviewed, and 107 (18%) were included for further analysis. There were 188 excluded because of duplicate exams: 236 because of technical limitations and 51 because of absent consent. A flow chart of the participant selection is shown in Figure 3.

The mean age was 61.8 ± 12.4 years; 46 participants were men (43%) and 61 were women (57%), and there were 76 right port accesses (71%) and 31 left (29%) (Table 1).

**Table 1.** Baseline demographic information.

*Demographics*		
Men, Women, *n* (%)	46 (43)	61 (57)
Age (years), mean ± SD	61.8 ± 12.4	
Access, left, right, *n* (%)	31 (29)	76 (71)

### Reliability

The ICCs were interpreted following the suggested values of Koo and Li^21^: values <0.5 are indicative of poor reliability, values between 0.5 and 0.75 indicate moderate reliability, values between 0.75 and 0.9 indicate good reliability, and values >0.90 indicate excellent reliability.

### Intraobserver comparisons

We found the following intraobserver agreement in both PJ and CS evaluations of DCAJ (continuous data).

The intraobserver agreement as indicated by the ICC was superior to 0.939 for normal (white bones) PJ imaging, equal to 0.945 for inverted (black bones) PJ imaging, and superior to 0.966 for CS imaging (Table 2). These values indicate excellent reliability.

**Table 2.** Intraobserver analysis.

Observer (comparisons)	Mean DCAJ difference ± SD	ICC [95% CI]
*Normal (white bone)*		
P11, P12	0.11 ± 0.58	0.957 [0.937-0.970]
P21, P22	−0.02 ± 0.76	0.939 [0.911-0.958]
C11, C12	0.10 ± 0.42	0.979 [0.969-0.986]
C13, C14	0.09 ± 0.54	0.966 [0.951-0.977]
*Inverted (black bones)*		
P13, P14	0.12 ± 0.69	0.945 [0.921-0.962]
P23, P24	0.03 ± 0.68	0.945 [0.920-0.962]
*Normal vs. Inverted*		
P1 mean N—mean IV	0.11 ± 0.46	0.986 [0.980-0.991]
P2 mean N—mean IV	−0.04 ± 0.76	0.965 [0.949-0.976]

Abbreviations: DCAJ = distance from tip to cavo-atrial junction; ICC = intraclass correlation coefficient; Normal (N) = image display with white bones; Inverted (IV) = image display with black bones; Pxx = Projectional observation/reader no./session no.; Cxx = Cross-sectional observation/reader no./session no.

The intraobserver agreement as indicated by the ICC was also superior to 0.965 for the comparison between normal PJ imaging (white bones) and inverted PJ imaging (black bones) (Table 2). The values indicated excellent reliability.

### Interobserver comparisons

We found the following agreement between the evaluations of DCAJ by the observers.

The interobserver agreement as indicated by the ICC was 0.936 for PJ imaging, and 0.987 for CS imaging (Table 3). These values indicate excellent reliability.

**Table 3.** Interobserver analysis.

Observer (comparisons)	Mean DCAJ difference ± SD	ICC [95% CI]	RC	*P* value
*Projectional imaging*				
P1 mean—P2 mean	0.19 ± 0.66	0.936 [0.906-0.956]	1.82	<.01
*Cross-sectional imaging*				
C1 mean—C2 mean	−0.24 ± 0.41	0.987 [0.981-0.991]	0.93	<.01
*Cross-sectional vs. Projectional*				
C mean—P mean	−0.13 ± 0.80	0.962 [0.944-0.974]	1.79	.100

Abbreviations: DCAJ = distance from tip to cavo-atrial junction; ICC = intraclass correlation coefficient; RCs = repeatability coefficients; Px = projectional observation/reader; Cx = cross sectional observation/reader.

Finally, the interobserver agreement as indicated by the ICC was 0.962 for PJ imaging and CS imaging (Table 3). This value indicates excellent reliability.

**Figure 4.**
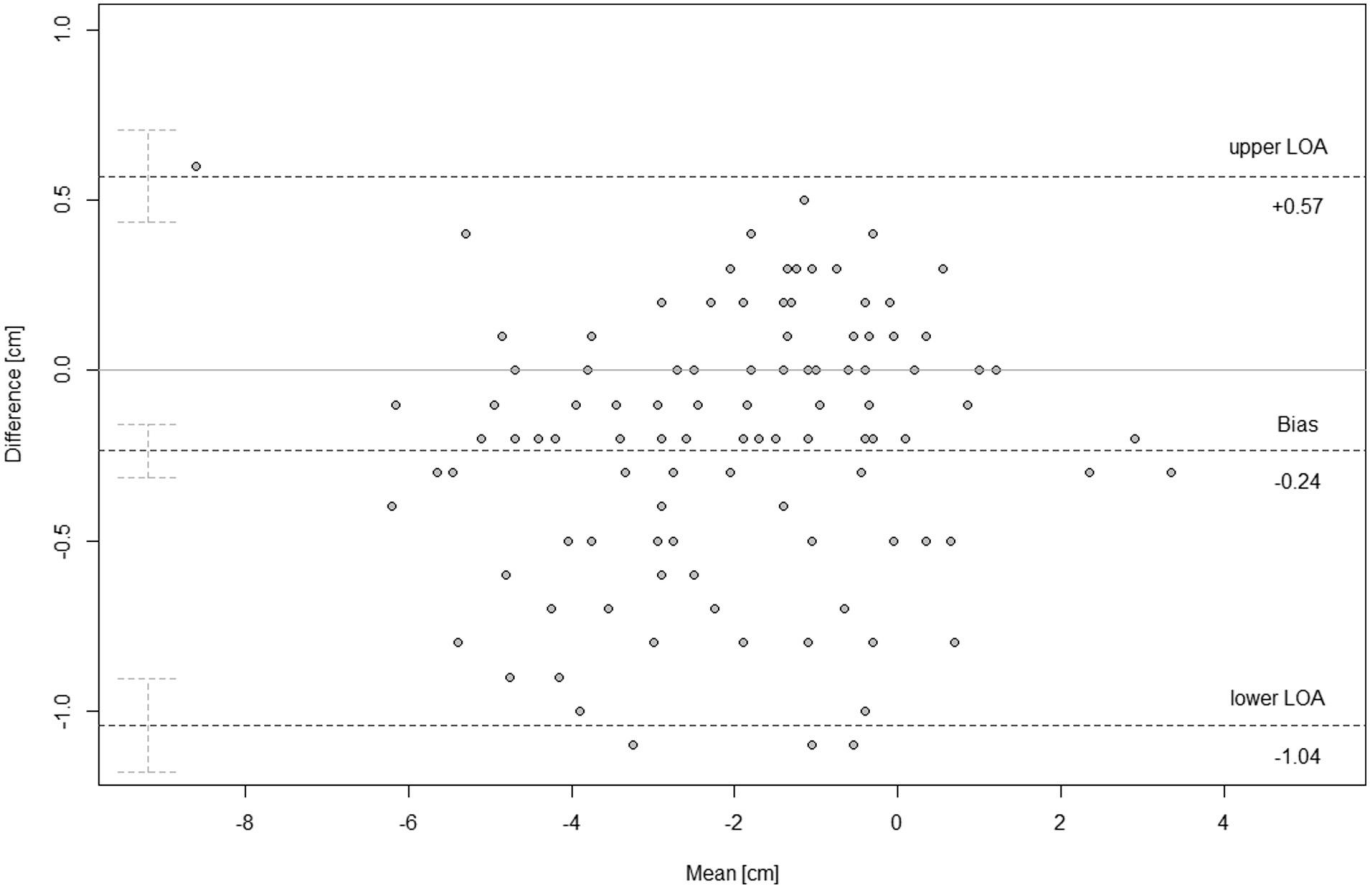
Bland-Altman plot showing cross-sectional (CS reader 1 mean) vs. cross-sectional (CS reader 2 mean) imaging interreader agreement (*N* = 107). Graphical display of the means against their respective paired differences. The Bland-Altman limits of agreement (upper/lower dashed lines) are displayed with their respective 95% confidence intervals. The mean of the differences (bias) is displayed as the central dashed line with the respective 95% confidence intervals. The zero *y* is the reference line of perfect average agreement.

The difference between paired readings of CS imaging (reader C1 mean vs. reader C2 mean) led to an estimated bias of −0.24 cm (*P* < .01) with a standard deviation of 0.41 cm and a precision between −0.32 cm and −0.16 cm. The LoA were 0.57 cm to −1.04 cm with outer 95% confidence limits of 0.71 cm and −1.17 cm. Repeatability coefficients equaled 0.93 cm (Figure 4, Table 3).

**Figure 5.**
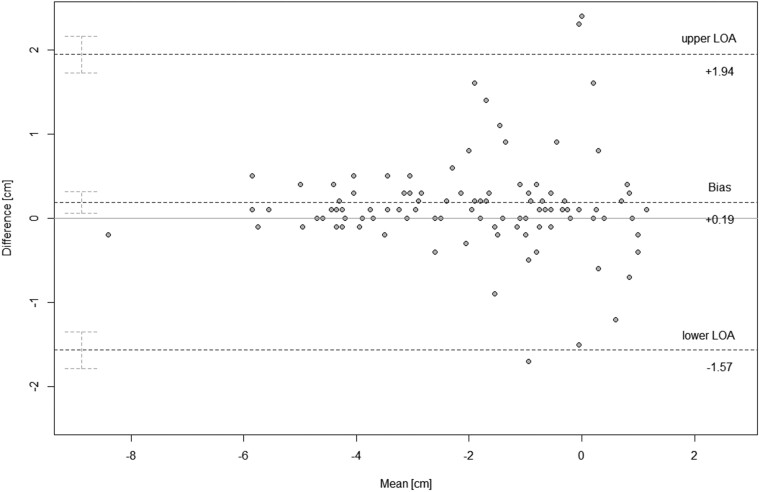
Bland-Altman plot showing projectional (PJ reader 1 mean) vs. projectional (PJ reader 2 mean) imaging interreader agreement (*N* = 107). Graphical display of the means against their respective paired differences. The Bland-Altman limits of agreement (upper/lower dashed lines) are displayed with their respective 95% confidence intervals. The mean of the differences (bias) is displayed as the central dashed line with the respective 95% confidence intervals. The zero *y* is the reference line of perfect average agreement.

The difference between paired readings of PJ imaging (reader P1 mean vs. reader P2 mean) led to an estimated bias of 0.19 cm (*P* < .01) with a standard deviation of 0.66 cm and a precision between 0.06 cm and 0.31 cm. The LoA were 1.94 cm to −1.57 cm with outer 95% confidence limits of 2.16 cm and −1.78 cm. Repeatability coefficients equaled 1.82 cm (Figure 5, Table 3).

**Figure 6.**
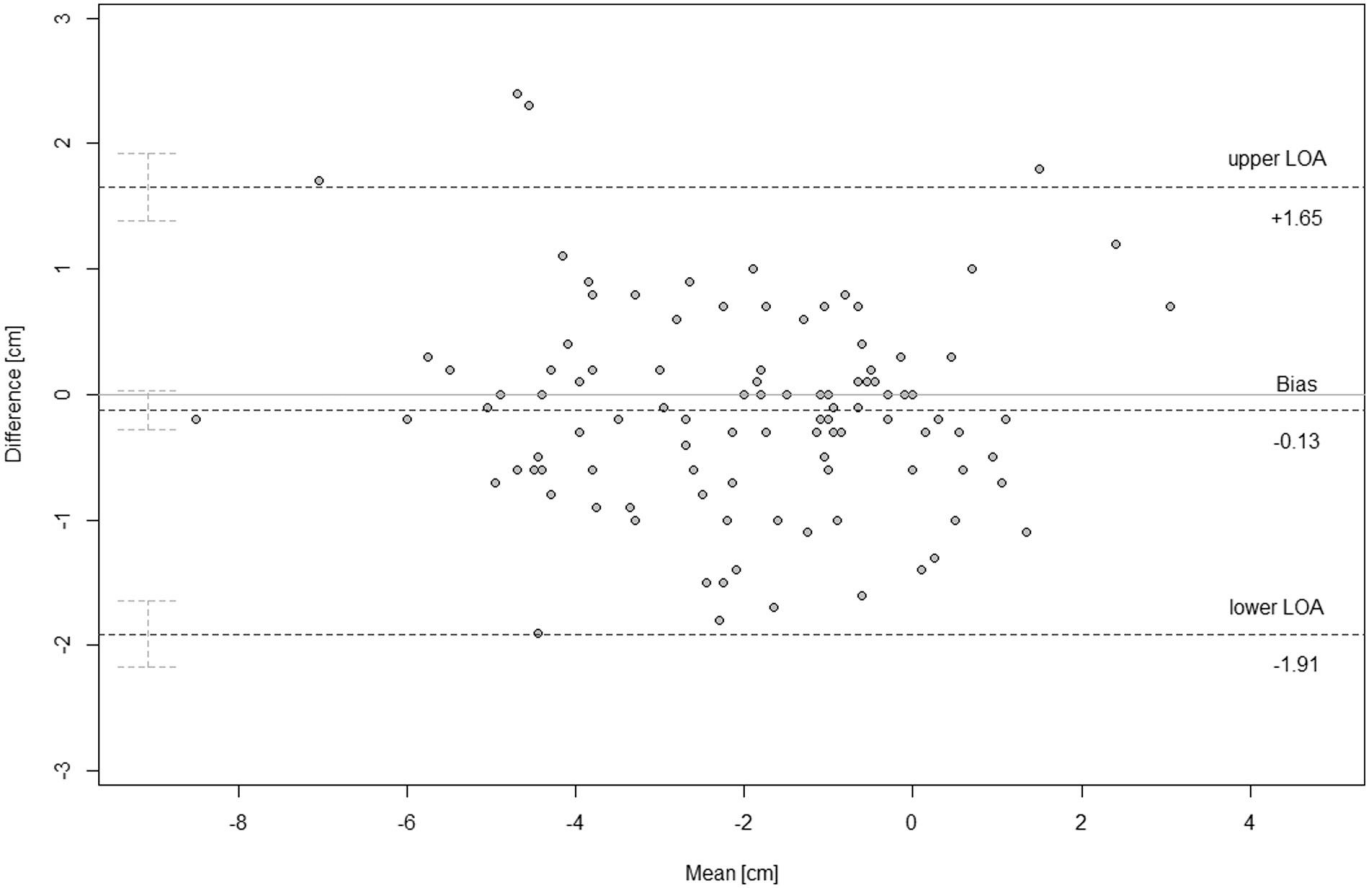
Bland-Altman plot showing cross-sectional (CS readers’ mean) vs. projectional (PJ readers’ mean) imaging interreader agreement (*N* = 107). Graphical display of the means against their respective paired differences. The Bland-Altman limits of agreement (upper/lower dashed lines) are displayed with their respective 95% confidence intervals. The mean of the differences (bias) is displayed as the central dashed line with the respective 95% confidence intervals. The zero *y* is the reference line of perfect average agreement.

The differences between paired readings of CS and PJ imaging (CS readers mean vs. PJ readers C2 mean) led to an estimated bias of −0.13 cm (*P* = .10) with a standard deviation of 0.80 cm and a precision between 0.03 cm and −0.28 cm. The LoA were 1.65 cm to −1.91 cm with outer 95% confidence limits of 1.92 cm and −2.17 cm. Repeatability coefficients equaled 1.79 cm (Figure 6, Table 3).

### DCAJ categorization

The number of optimal, suboptimal, and needing repositioning tip locations were respectively 39, 35, and 33 for PJ and 33, 41, and 33 for CS. The matching of categories (pairwise comparison) fails 29% of the time. Perfect matches represent roughly 25% for T1, T2, and T3 categories (Table 4).

**Table 4.** Pairwise comparison.

PJ/CS	T1 CS	T2 CS	T3 CS
**T1 PJ**	**26**	**13**	**0**
	0.24	0.12	
**T2 PJ**	**7**	**23**	**5**
	0.07	0.21	0.05
**T3 PJ**	**0**	**5**	**28**
		0.05	0.26

Mean DCAJ (Distance from tip to cavo-atrial junction) subdivided into categories for Projectional observers (PJ) and Cross-sectional observers (CS).

T1: tip of the catheter within 1 cm of the cavo atrial junction (CAJ). T2: tip of the catheter within 1-3 cm of the CAJ. T3: tip of the catheter more than 3 cm distant from the CAJ.

Results are displayed as numerical values (bold number - upper cell) and proportional values (decimal number - lower cell).

## Discussion

The imaging modality usually accepted as the standard of care for assessing CVC tip position is CXR, given its wide availability and relatively low radiation dose. To our knowledge, this is the first study to question the reliability of CXR in defining CVC tip position compared to the gold standard of CT, while also evaluating observer variability in consistency and accuracy.

In PAC placement, radiology and surgery dominate, with radiology accounting for approximately 30% of cases and surgery for 70% since 2011.^22–24^ This trend persists despite the higher costs associated with using operating rooms for surgical placement.^25^

Radiologists typically use ultrasound for vein access and fluoroscopy for tip position confirmation. In contrast, surgical approaches vary, sometimes involving ultrasound, fluoroscopy, or blind guidance, followed by routine CXR confirmation.^22^^,^^26^ Consequently, CXR remains a widely utilized tool in PAC placement worldwide.^22^^,^^26^

In ICU settings, CXR is still considered appropriate for CVC insertions, with a favorable risk-benefit ratio for patients.^27^ Furthermore, CXR plays a central role in evaluating catheter position, and its importance is expected to grow with the advent of artificial intelligence solutions designed to enhance detection of positioning errors, prevent complications, and identify pneumothorax.^28^^,^^29^

### Intra- and inter-observer variability

In our study, intra-observer reproducibility of DCAJ was consistent with the 0.917 reported by Chan et al^30^ suggesting that CXR can effectively differentiate DCAJ variations between patients. Interobserver ICC was superior to the 0.355 observed by Chan et al^30^ likely due to their high number of readers (13). Our ICC was closer to the 0.833 obtained by So et al.^31^

The ground theory of eye detection suggests a 20% difference in thresholds between negative and positive stimuli,^32^ indicating that observers were more comfortable in identifying CVC on black bone images, similar to the results of Kheddache et al.^33^ Moreover, Sun et al. reported an improvement in ICC of 0.05-0.06 when switching from regular to inverted images in their spinopelvic sagittal vertical axis study.^34^

The results of our comparison between white bone and inverted black bone images were mixed. One observer showed minimal improvement with black bone imaging, while the other had better results with white bone images. We attribute this difference to individual variability, such as cognitive training or familiarity with black bone imaging, which can either enhance performance with grayscale inversion^35^ or have no significant advantage.^36^

### RC and tip position uncertainty

Interobserver RC was in the same range as the 2.2 cm reported by Chan et al.^30^ On CT, tip location uncertainty on CS was nearly 1 cm, reflecting solely the uncertainty in identifying the CAJ. Thus, variations smaller than 1 cm should not be considered real changes when reading CT images and presumably represent the absolute inferior limit for PJ. On PJ, the RC was approximately 2 cm, representing inaccuracy in both CAJ identification and tip location. Due to the superposition of structures, projectional images cannot be superior to the results of CS mentioned earlier; thus, at least 1 cm should be attributed to CAJ identification. The remaining (up to 1 cm) may be attributed to variations in tip identification. The combination of CAJ identification and tip location uncertainty leads to variability of up to 2 cm, suggesting that variations under 2 cm may not be reliable indicators of change in PJ readings.

The difficulty in localizing the catheter tip on CXR is linked to the low contrast in the mediastinum, which may sometimes blur the tip of the catheter. Enhancing catheter visibility, such as through contrast media injection, could improve tip confirmation by about 20%^37^ as well as the readers’ agreement.^38^ Likewise, digital CXR processing algorithms designed to enhance catheter outlining could also reduce RC variability.^39^ Edge enhancement and fractional multiscale image processing proved to increase catheter conspicuity.^40^^,^^41^

### PJ vs. CS imaging comparison

On average, tip location on PJ images was measured 0.13 cm more caudal than on CS, but this difference was not statistically significant (*P* = .10). As the average was close to zero this suggests that the variability between PJ and CS imaging is primarily due to measurement imprecision. Previous studies have related a structural difference of 10-18 mm between CAJ positions identified on CT vs. PJ,^31^^,^^42^^,^^43^ which may be attributed to the different anatomic landmarks used: crista terminalis on CT vs. the cardiac silhouette on PJ.

Our study found a RC equal to almost 1.8 cm when comparing PJ and CS imaging; it cannot be ruled out that the differences noted in the afermentioned studies may be instead due to individual observer variation. A similar trend has been found in ultrasound and CT comparison of kidney length, where the 95% CI ranged from −1.3 cm to 1 cm. The author attributed the difference to measurement imprecision (ie, individual variations) rather than structural variation.^44^ Further studies are needed to investigate this alternative interpretation.

### Management of potential complications

Complications from CVC tip malpositioning, despite confirmation on CXR, are challenging to document comprehensively. Overinsertions, for example, can cause mechanical irritation of the endocardium, leading to advanced ventricular dysrhythmias in up to 10% of cases.^45^ However, due to continuous cardiac monitoring, rapid catheter withdrawal typically makes this complication a self-limited per-procedural event^45^^,^^46^ and is, therefore, rarely documented.

A CVC tip placed at the CAJ allows balancing mechanical, organic,^47^^,^^48^ and cardiac complications. Positioning the tip within 3 cm zone of the CAJ is considered an acceptable target.^5^^,^^14^^,^^49^ Beyond this cutoff, catheters are not acceptable in terms of accuracy and are deemed to be repositioned.

**Figure 7.**
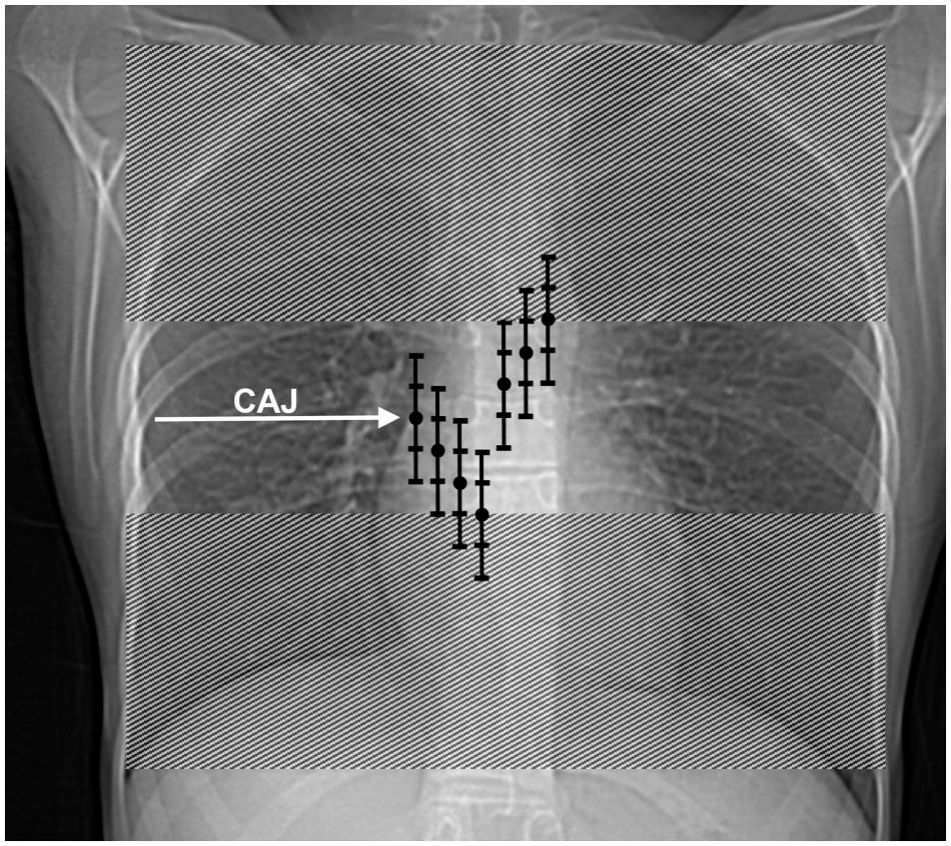
Tip anatomical positions with observers’ variability. CAJ: cavo-atrial junction. Dot: anatomical position of the tip. Ladder: ±2 cm observer variation. Dashed lines: non acceptable tip positioning zone. From left to right: tip position at CAJ, 1 cm beyond CAJ, 2 cm beyond CAJ, 3 cm beyond CAJ, 1 cm before CAJ, 2 cm before CAJ, 3 cm before CAJ.

However, PJ-based tip position assessments have a ±2 cm variability, meaning that to ensure the tip is within the acceptable anatomical zone, it must be measured within 1 cm of the CAJ on PJ. In other words, a reading of the tip position that deviates by more than 1 cm on CXR may indicate an anatomical position outside of the acceptable zone of ±3 cm from the CAJ. Therefore, we recommend using PJ-based readings of ±1 cm as a threshold for acceptable tip positioning (Figure 7).

Reducing the acceptable PJ-based threshold to 1 cm could lead to reclassifying 30% of catheters (usually considered suboptimal) as inadequate, needing repositioning.^5^

### Limitations

This study has several limitations. First, topograms may have lower resolution than CXR, impacting catheter detection and observer reliability.^31^ However, topograms benefit from perpendicular photon alignment, reducing parallax errors associated with CXR.^50^

Second, nongated CT imaging introduced heart motion artifacts contributing to CAJ identification uncertainty. Third, the luminosity, contrast, and magnification used were observer-specific and may have influenced results. Lastly, our study focused on port catheters, but the malpositioning rate was similar to that reported by Glauser et al. for bedside placements.^49^

## Conclusion

CXR as well CT assessment of CVC tip position are subject to both intra-individual and inter-individual variability. While on CT it is associated to CAJ determination only, on CRX it is linked to both CAJ and catheter tip determination, leading to a variability of approximately 2 cm.

Although a 3 cm anatomical zone around the CAJ is generally accepted, the inherent 2 cm variability in CXR interpretation suggests reducing the acceptable CXR-based threshold to 1 cm. Despite its limitations, CXR remains a reliable method for determining CVC positioning.
